# C9orf72 mutation is rare in Alzheimer's disease, Parkinson's disease, and essential tremor in China

**DOI:** 10.3389/fncel.2013.00164

**Published:** 2013-09-24

**Authors:** Bin Jiao, Ji-feng Guo, Ya-qin Wang, Xin-xiang Yan, Lin Zhou, Xiao-yan Liu, Fu-feng Zhang, Ya-fang Zhou, Kun Xia, Bei-sha Tang, Lu Shen

**Affiliations:** ^1^Department of Neurology, Xiangya Hospital, Central South UniversityChangsha, China; ^2^State Key Laboratory of Medical GeneticsChangsha, China; ^3^Key Laboratory of Hunan Province in Neurodegenerative Disorders, Central South UniversityChangsha, China

**Keywords:** *C9orf72*, Alzheimer's disease, Parkinson's disease, essential tremor, risk factor

## Abstract

GGGGCC repeat expansions in the *C9orf72* gene have been identified as a major contributing factor in patients with amyotrophic lateral sclerosis (ALS) and frontotemporal dementia (FTD). Given the overlapping of clinical phenotypes and pathological characteristics between these two diseases and Alzheimer's disease (AD), Parkinson's disease (PD), and essential tremor (ET), we speculated regarding whether *C9orf72* repeat expansions also play a major role in these three diseases. Using the repeat-primed polymerase chain reaction method, we screened for *C9orf72* in three groups of patients with PD (*n* = 911), AD (*n* = 279), and ET (*n* = 152) in the Chinese Han population. There were no pathogenic repeats (>30 repeats) detected in either the patients or controls (*n* = 314), which indicated that the pathogenic expansions of *C9orf72* might be rare in these three diseases. However, the analysis of the association between the number of repeats (*p* = 0.001), short/intermediate genotype (short: <7 repeats; intermediate: ≥7 repeats) (odds ratio 1.37 [1.05, 1.79]), intermediate/intermediate genotype (Odds ratio 2.03 [1.17, 3.54]), and PD risks indicated that intermediate repeat alleles could act as contributors to PD. To the best of our knowledge, this study is the first to reveal the correlation between *C9orf72* and Chinese PD, AD, or ET patients. Additionally, the results of this study suggest the novel idea that the intermediate repeat allele in *C9orf72* is most likely a risk factor for PD.

## Introduction

A hexanucleotide (GGGGCC) repeat expansion in the first intron of the *C9orf72* gene was recently identified as a major contributing factor to the chromosome 9p21-linked diseases amyotrophic lateral sclerosis (ALS) and frontotemporal dementia (FTD) (Dejesus-Hernandez et al., 2011). As previously reported, the *C9orf72* mutation accounts for 23.5–47% of familial ALS/FTD and 4.1–21.0% of sporadic ALS in white populations (Dejesus-Hernandez et al., 2011; Renton et al., 2011; Gijselinck et al., 2012). The pathogenic mechanism of repeat expansions primarily includes interference with the normal expression of the encoded protein or the loss of protein function through the generation of abnormal toxic RNA foci that disrupt normal cellular pathways (Renton et al., 2011; Sha and Boxer, 2012).

There is no doubt that the overlapping presentations of clinical phenotypes, pathological characteristics, and gene mutations exist among Parkinson disease (PD), Alzheimer's disease (AD), and ALS/FTD (Hudson, 1981; Piguet et al., 2011; Arighi et al., 2012; Floris et al., 2012; O'Dowd et al., 2012). First, in the examination of clinical phenotypes, relatives of patients with ALS have an increased risk for developing PD and AD, additionally, some ALS/FTD patients have developed the associated features of Parkinsonism and movement disorders (Hsiung et al., 2012; Takada et al., 2012; Kohli et al., 2013). Second, the presence of TAR DNA-binding protein-43(+) intranuclear inclusions, which are the pathological feature of chromosome 9p21-linked ALS/FTD, have been detected in PD and AD patients (Nakashima-Yasuda et al., 2007; Boeve et al., 2012). Finally, mutations in the microtubule-associated protein tau (MAPT) gene could cause a spectrum of phenotypes which include ALS, Parkinsonism, and cognitive impairment (O'Dowd et al., 2012). Given the considerations above, one question has yet to be addressed. Could the *C9orf72* repeat expansions account for other neurodegenerative disorders, such as AD, PD, and essential tremor (ET)?

In *C9orf72*, Repeat expansions exceeding 30 units have been suggested to be pathological in ALS/FTD patients (Dejesus-Hernandez et al., 2011). Interestingly, pathogenic expansions have also been observed in patients with PD, AD, progressive supranuclear palsy, corticobasal degeneration, and Lewy body dementia (Xi et al., 2012; Cacace et al., 2013; Lesage et al., 2013), which further indicates that the phenotypes that are associated with repeat expansions could include the spectrum of cognitive impairment and movement disorder syndromes. In addition, a previous study has demonstrated that the role of intermediate repeats (7–24 repeat units) is strongly associated with these diseases and the expression of *C9orf72*. The significantly decreased transcriptional activity of *C9orf72* with an increasing number of normal repeats indicates that intermediate repeats may act as predisposing alleles and favors the loss-of-function disease mechanism (Van Der Zee et al., 2013).

In this study, we first assess the prevalence of *C9orf72* repeat expansions in a large cohort of Chinese Han patients with AD, PD, or ET to determine whether repeat expansions play a role in these three common disorders. Furthermore, we explore whether repeat expansions of intermediate repeats might be a risk factor for AD, PD, or ET, and/or could affect the age at onset in patients with these three diseases.

## Materials and methods

### Study samples

Three independent series of patients participated in this study: the first cohort of 911 sporadic PD patients that met the UK brain bank diagnosis criteria (Hughes et al., 1992); the second cohort of 279 sporadic AD patients that met the NINCDS-ADRDA criteria for probable or definite AD (McKhann et al., 1984); and the third cohort of 152 ET patients that met the Washington Heights-Inwood Genetic Study of ET (WHIGET) diagnosis criteria (Louis et al., 1997). All patients were recruited from the outpatient neurology clinics of the Xiangya Hospital, Central South University. In total, 314 healthy Chinese individuals were recruited from the Xiangya Wellness Center as a control group. Informed consents for participation in the study were obtained from all subjects, including patients and controls. This study received prior approval by the institutional review board and the ethics committee of the Xiangya Hospital, Central South University.

### Methods

Genomic DNA was isolated from peripheral blood leukocytes using a QIAGEN kit. We screened the presence of the GGGGCC hexanucleotide expansion of *C9orf72* using a 2-step polymerase chain reaction protocol. In the first step, we used a previously reported repeat-primed polymerase chain reaction assay to detect the size of the larger expanded alleles (Dejesus-Hernandez et al., 2011). Briefly, DNA samples (50 ng/μ l) were amplified using three primers (MRX-F: 5′FAM-ACAGTACTCGCTGAGGGTGAAC; MRX-R1: 5′CAGGAAACAGCTATGACCGGGCCCGCCCCGACCACGCCCCGGCCCCGGCCCCGG; MRX-M13R: 5′CAAGGAAACAGCTATGACC), and the primers ratio (0.6 μ l of 10 μ M of MRX-F; 0.6 μ l of 10 μ M of MRX-M13R; 0.1 μ l of 10 μ M of MRX-R1) were modified to improve the efficiency of the PCR. Other components of the PCR reaction included the following: 1_μ l of 50_ng/μ l of DNA samples, 2_μ l of 5× Q-solution (QIAGEN Valencia, CA, USA), 0.7 μ l of 100% DMSO (Sigma-Aldrich), 0.36 μ l of 7-deaza-dGTP (New England Biolabs, Ipswich, MA, USA), 0.2 μ l of Roche FastStart Taq DNA polymerase, 0.2 μ l of 10 μ M dNTP and 1 μ l of 10× Buffer (Roche Applied Science, Indianapolis, IN, USA), and 3.24 μ l MQ. The total process was performed using a touchdown thermocycling program. The reaction conditions consisted of 95°C for 5 min, 15 cycles of 95°C for 1 min, 70°C for 1 min, with a decrement of 1°C per cycle, 72°C for 3 min, followed by 25 cycles of 95°C for 1 min, 56°C for 1 min, 72°C for 3 min, 72°C for 60 min, the final temperature was then sustained at 15°C. In the second step, we performed a classical FAM-fluorescent labeled PCR assay to detect the accurate genotype of the non-pathogenic mutation carriers. The fragment length analysis was performed on an ABI 3730×l DNA analyzer and was visualized by the GeneMapper software version 3.2 (Applied Biosystems).

### Statistical analysis

A cut-off value of 30 repeats was used to define the pathogenic threshold (Renton et al., 2011). One DNA sample of an ALS patient who was recruited from the Xiangya Hospital was subjected to a repeat-primed polymerase chain reaction, and >30 repeat expansions were detected (unpublished paper), which could verify the reliability and trustworthiness of our experiment. Descriptive statistics were expressed as the mean ± the standard deviation; differences in the distributions of repeat number between the patients and controls were tested using a 2-tailed Mann-Whitney U-test or Kruskal–Wallis H-test, and significance was set at *p* = 0.05. Considering that the role of ≥7 units in non-pathogenic carriers was strongly correlated with *C9orf72* expression (Van Der Zee et al., 2013), all subjects were classified into three genotypes, including S/S, S/I, and I/I (S: short allele <7 units; I: intermediate allele ≥7_units) according to an individual's two repeat alleles. The associations between the number of repeat and disease risk were determined in logistic regression models that were adjusted for the age at onset and gender. Analyses of the associations between the number of repeats and the age at onset were calculated using linear regression models that were adjusted for gender. To adjust *p*-values for multiple testing, we performed Bonferroni adjustment in logistic regression and linear regression. The statistical analysis was performed using the SPSS program (version 18.0).

## Results

**Figure 1:**
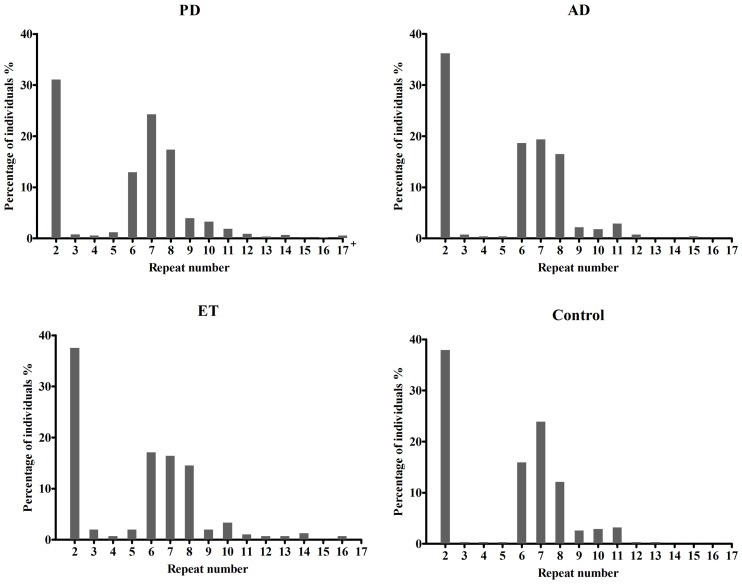
**Distributions of repeat number in C9orf72 in Alzheimer's disease, Parkinson disease, essential tremor, and control individuals**. There was no significant difference in the distributions of repeat length between AD (*p* = 0.67), ET (*p* = 0.13) cases and controls, respectively. However, an evidence of significant distribution was identified between PD cases and controls (*p* = 0.01); 17^+^ in PD patients including five PD cases at 18, 19, 22, 23, 27 repeats.

Table 1 presents the demographic information for our study. A total of 1342 patients and 314 healthy controls were successfully subjected to repeat-primed polymerase chain reactions and genotyping. However, no pathological repeat expansion of *C9orf72* was detected in either patients or controls. The wide range of repeat expansions in patients was 2–27 units, and the most frequent repeats in all subjects was 2 units, followed by 6, 7, and 8; however, three PD patients harbored marginally larger alleles at 22, 23, and 27 units. In addition, the distributions of repeat numbers in the individuals' larger allele indicated a significant difference in PD (*p* = 0.01), late onset PD (>50 years) (*p* = 0.01), and male PD (*p* = 0.03) when compared with controls. However, no statistical significance was found in AD (*p* = 0.67), ET (*p* = 0.13) or their subtypes when compared with control individuals (Figure 1 and Table 2).

**Table 1:** **Demographic information and average number of repeats among cases and controls**.

**Variable**	**PD**	**AD**	**ET**	**Controls**
Cases, *n*	911	279	152	314
Gender, Male (%)	533 (58.5)	167 (59.9)	89 (58.6)	158 (50.3)
Age	59.0 ± 11.6	72.4 ± 12.0	44.7 ± 15.8	57.1 ± 13.7
Age at onset	55.1 ± 11.7	70.1 ± 10.0	35.4 ± 14.9	–
Repeat number	5.9 ± 3.2	5.4 ± 2.9	5.4 ± 3.1	5.3 ± 2.9

The sample mean ± SD is given for age, age at onset, and repeat number.

**Table 2:** **Analysis of the distributions of repeat expansion in cases with different classification**.

	**EOAD vs. Control**			**LOAD vs. Control**		
	***N***	**Mean ± *SD***	***P***	***N***	**Mean ± *SD***	***P***
Cases	71	5.5 ± 2.8	0.86	208	5.4 ± 3.0	0.77
Controls	230	5.3 ± 2.9		84	5.3 ± 2.9	
	**Male in AD vs. Control**			**Female in AD vs. Control**		
Cases	106	5.7 ± 2.9	0.36	173	5.3 ± 2.9	0.77
Controls	158	5.2 ± 2.9		156	5.4 ± 2.8	
	**EOPD vs. Control**			**LOPD vs. Control**		
Cases	286	5.6 ± 2.0	0.19	603	6.0 ± 3.2	0.01
Controls	85	5.2 ± 2.7		229	5.4 ± 2.9	
	**Male in PD vs. Control**			**Female in PD vs. Control**		
Cases	533	5.9 ± 3.0	0.03	378	5.9 ± 3.3	0.07
Controls	158	5.3 ± 2.9		156	5.4 ± 2.8	
	**Male in ET vs. Control**			**Female in ET vs. Control**		
Cases	89	5.5 ± 3.2	0.61	63	5.2 ± 2.9	0.57
Controls	158	5.2 ± 2.9		156	5.4 ± 2.8	

EOAD, early onset AD (≤65 years); LOAD, late onset AD (>65 years); EOPD, early onset PD (≤50 years); LOPD, late onset PD (>50 years).

Given the significant distinction of repeat length in PD and control individuals, 202 PD patients who completed a battery of neuropsychological tests that were recommended by the Movement Disorder Society (MDS) Task Force, were further divided into 70 PD dementia (PD-D), 58 PD mild cognitive impairment (PD-MCI), and 74 PD with no cognitive impairment (PD-NC) according to the MDS Task Force diagnosis criteria(Dubois et al., 2007). However, no significant difference in the distribution of repeats was found among the three subgroups and control individuals (*p* = 0.98) using Kruskal–Wallis H-test.

In addition, we employed two analytical approaches for association testing, the larger repeat allele as a continuous variable and three genotyping categorical variables based on the individual's short or intermediate alleles. We did not identify any significant evidence of associations between the number of repeat and either the AD or ET risk or the age at onset in patients with PD or ET. Interestingly, we observed a statistically significant result between the I/I genotype and the age at onset (*p* = 0.007; Regression coefficient 5.50 [0.27, 10.74]) after Bonferroni adjustment for multiple testing, but not in the continuous variable (*p* = 0.93) (Table 3). Finally, the only significant evidence of association was found between the repeat length and PD risk after using Bonferroni adjustment when considering repeats as a continuous variable (*p* = 0.001, OR 1.06 [1.01, 1.10]), or when considering three genotypes as a categorical variable (*p* = 0.0081; S/I: OR 1.37 [1.05, 1.79]; I/I: OR 2.03 [1.17, 3.54]) (Table 4).

**Table 3:** **Association of allele length with age at onset in patients with PD, AD, and ET**.

	**Association of allele length with age at onset**					
	**PD (*n* = 911)**		**AD (*n* = 279)**		**ET (*n* = 152)**	
	**Regression coefficient (95% CI)**	***P***	**Regression coefficient (95% CI)**	***P***	**Regression coefficient (95% CI)**	***P***
**Genotype**		0.43		0.007		0.85
S/S	0.00 (reference)		0.00 (reference)		0.00 (reference)	
S/I	0.02 (−1.60, 1.63)		1.04 (−1.90, 3.98)		−0.15 (−3.85, 3.55)	
I/I	1.37 (−1.41, 4.16)		5.50 (0.27, 10.74)		−0.32 (−7.57, 6.93)	
**Repeats on larger allele continuous**						
	0.20 (−0.48, −0.44)	0.12	0.02 (−0.46, 0.50)	0.93	−0.47 (−1.20, 0.25)	0.20

CI, confidence interval; S, short allele (<7 units); I, intermediate allele (≥7 units).

Regression coefficient and corresponding p results from linear regression models adjusted for gender. After Bonferroni adjustment for multiple testing, p ≤ 0.025 were considered statistically significant (2 tests).

**Table 4:** **Association of allele length with risk of PD, AD, and ET diseases**.

	**Association of allele length with risk of disease in comparison with controls (*n* = 314)**					
	**PD (*n* = 911)**		**AD (*n* = 279)**		**ET (*n* = 152)**	
	***OR* (95% CI)**	***P***	***OR* (95% CI)**	***P***	***OR* (95% CI)**	***P***
**Genotype**		0.0081		0.54		0.64
S/S	1.00 (reference)		1.00 (reference)		1.00 (reference)	
S/I	1.37 (1.05, 1.79)		0.83 (0.55, 1.26)		0.88 (0.57, 1.37)	
I/I	2.03 (1.17, 3.54)		0.91 (0.40, 2.06)		1.30 (0.56, 3.04)	
**Repeats on larger allele continuous**						
	1.06 (1.01, 1.10)	0.001	0.99 (0.93, 1.05)	0.69	0.96 (0.89, 1.03)	0.27

OR, odds ratio; CI, confidence interval; S, short allele (<7 units); I, intermediate allele (≥7 units).

Odds ratios and corresponding p-value results from logistic regression models adjusted for age at onset and gender. After Bonferroni adjustment for multiple testing, p ≤ 0.0083 were considered statistically significant (6 tests).

## Discussion

Recently, GGGGCC repeat expansions in the *C9orf72* gene were identified as major contributing factors for ALS and FTD. However, the preliminary evidence suggested that the *C9orf72* mutation rates in patients with clinically diagnosed ALS in China, Japan, Korea, and Taiwan were much lower than that observed in Caucasian populations (Ogaki et al., 2012; Tsai et al., 2012; Zou et al., 2012; Jang et al., 2013), which implied that the number of repeats varied greatly due to different nationalities and ethnicities. Given the clinical heterogeneity with the repeat expansions, we hypothesized that the length of repeats may also account for other neurodegenerative disorders. To investigate the hexanucleotide repeat expansions of *C9orf72* on different genetic backgrounds, we screened for *C9orf72* in a large group AD, PD, and ET patients with Chinese Han origin. To the best of our knowledge, this study is the first reported investigation of *C9orf72* repeat expansions in three cohorts of patients in Asia.

In this study, no pathogenic expansion was observed in either patients or controls, which supported recent data from other independent cohorts. Across these studies, no abnormal repeats were found in 781 patients with PD and 568 patients with AD (Majounie et al., 2012a; Rollinson et al., 2012). The relation between AD, PD, and *C9orf72* has been controversial. Several studies have reported that pathogenic repeats were found in 0.7% patients with PD and in less than 1% of clinically diagnosed AD patients (Majounie et al., 2012b; Xi et al., 2012). However, Majounie et al. speculated that the positive results in AD patients might be an incidental rather than a causative finding due to amnesic FTD being misdiagnosed as probable AD (Majounie et al., 2012b).

There are three possible explanations for our negative results. The first possibility is that the hexanucleotide repeat expansions could not cause PD, AD, or ET, and is only specific to ALS/FTD. Although the pathogenic gene mutations were detected in several probable cases of PD or AD, the exact diagnoses of these diseases should be confirmed due to clinical heterogeneity. Another explanation for the result is that the cut-off value of repeat >30 units that is suitable for ALS/FTD is most likely not suitable for AD, PD, or ET patients, and more samples should be included to set a solid cut-off value. Finally, Mok et al. found that the ALS/FTD patients with *C9orf72* pathogenic repeats share a similar risk haplotype with Finland, Ireland, Italy, UK, and USA populations. Moreover, an investigation from Japan suggests that the pathogenic expansion is closely tied to the risk haplotype, and the low frequency of the risk haplotype might explain the low frequency of repeat expansions (Konno et al., 2012).

Although no pathogenic expansion was observed in this study, we identified a significant association between the number of intermediate repeats and PD risk, which indicates that the more intermediate repeats, the greater risk of susceptibility to PD. As we know, this study is the first to raise this notion. This notion suggests that the intermediate alleles act as a contributor to PD risk. Although our research to elucidate the disease mechanism of this intermediate repeat remains in its infancy, one previous study has indicated that intermediate repeats could decrease the transcriptional activity of *C9orf72* (Van Der Zee et al., 2013). Therefore, how the intermediate alleles act as predisposing alleles and what is the pathogenic pathway of intermediate alleles in PD patients should be addressed in further studies. In addition, this study reported that there was no significant difference in repeat expansions among PDD, PD-MCI, PD-NC patients, and control individuals, which indicated that the number of repeats in *C9orf72* might not account for the occurrence and severity of cognitive syndromes in PD patients.

Recently, Kohli et al. (2013) found nine AD patients that carried the *C9orf72* mutation with an average disease onset of 77.8 years (all older than 60 years), and speculated that the pathogenic repeat expansions were most likely associated with late onset AD (>65 years). Similarly, in this study, we observed a positive correlation between the I/I genotype and the age at onset, which indicated that AD patients who carried I/I genotypes were susceptible to a higher age at onset. At present, *APOE4* is the only major genetic risk factor for the development of late onset AD (Hauser and Ryan, 2013), if the relation above exists, then future research will focus on exploring whether there is an interaction mechanism between these two genes in patients with late onset AD. Finally, there was only a cohort of 106 ET patients that were investigated before our study, and this cohort had the identical result as our study, with no association of normal repeat length with disease risk or with an effect on age at onset, further confirming that GGGGCC repeats did not play a role in patients with ET.

However, there are several limitations in this work. First, an association between the I/I genotype and the age at onset in AD patients might be a false positive observation due to the lack of a statistically significance correlation between the larger alleles (continuous variables) and the age at onset. Therefore, we should recruit more samples to verify the relation between the repeat expansions in *C9orf72* and the age at onset in patients with AD. Second, if the relation above was further verified, then we should assess the relation between the *C9orf72* repeats and *APOE* genotypes to explore whether there is an interaction mechanism between them. Third, this study only examined sporadic AD, PD, and ET patients, *C9orf72* may play a role in dominant familial forms.

In conclusion, no *C9orf72* pathogenic mutations in AD, PD, and ET patients in this study are consistent with previous studies (Majounie et al., 2012b; Dejesus-Hernandez et al., 2013). However, we identified a statistically significant association between the intermediate repeats and PD risk, which implied that intermediate genotypes or alleles might be a risk factor for PD in China. Meanwhile, the lack of association between the intermediate repeats and AD risk has indicated that intermediate genotypes or alleles might play a different role in AD and PD.

## Author contributions

Lu Shen, Beisha Tang, and Kun Xia contributed to the conception and organization of our research project; Bin Jiao, Jifeng Guo, Xinxiang Yan, and Lin Zhou contributed to recruiting patients and controls; Bin Jiao, Jifeng Guo, Yaqin Wang, Xiaoyan Liu, Fufeng Zhang, and Yafang Zhou contributed to statistical analysis; Bin Jiao and Jifeng Guo equally contributed to the first draft, and Lu Shen was responsible for the review and critique of our manuscript.

### Conflict of interest statement

The authors declare that the research was conducted in the absence of any commercial or financial relationships that could be construed as a potential conflict of interest.
